# Clinical Perspectives of Mesenchymal Stem Cells

**DOI:** 10.1155/2012/684827

**Published:** 2012-12-24

**Authors:** Jan Kramer, Francesco Dazzi, Massimo Dominici, Peter Schlenke, Wolfgang Wagner

**Affiliations:** ^1^Interdisciplinary Group Stem Cell Biology and Transplantation Unit, Medical Department I, University of Lübeck, Ratzeburger Allee 160, 23538 Lübeck, Germany; ^2^Stem Cell Biology, Department of Medicine, Imperial College London, London SW7 2AZ, UK; ^3^Division of Oncology, University of Modena and Reggio Emilia, 41100 Modena, Italy; ^4^Institute of Transfusion Medicine and Transplantation Immunology, University of Münster, Münster, Germany; ^5^Stem Cell Biology and Cellular Engineering, Helmholtz Institute for Biomedical Technology, RWTH Aachen University Medical School, 52074 Aachen, Germany

Mesenchymal stem cells (MSCs) raise high hopes for regenerative medicine—in fact, they have already paved their way to a large number of clinical trials for a broad range of diseases. So far, several studies provided promising results, but this relatively new area of research requires further validation as some of the studies revealed varying outcomes. This might be a result of the heterogeneity of MSC cultures and absence of reliable protocols for isolation of the naïve stem cell fraction. MSCs are not precisely defined on a molecular level. They can be isolated from many tissues under different culture conditions—yet, they comprise multiple subpopulations and only a subset reveals multipotent differentiation capacity into at least adipogenic, chondrogenic, and osteogenic lineages. Notably, the composition of subpopulations seems to be greatly affected by culture methods and in the course of culture expansion. 

Culture of MSCs has already been established in the early 1960s when fibroblastoid cells were discussed as supportive stromal cells within the hematopoietic bone marrow niche. Initially, application of MSCs in regenerative settings was mainly based on the hope to cure diseases or defects of cartilage, bone, or adipogenic tissue. Their use for musculoskeletal diseases still remains one of the most frequent applications (Figure 1). Particularly in an autologous setting, differentiated derivatives of MSCs may be functionally integrated in constructs to enhance regeneration of bone or cartilage defects.

There is a growing perception that MSCs reveal additional attributes which open further clinical perspectives: they seem to secrete active molecules which are capable to stimulate regeneration. The precise nature of these molecules, for example, growth factors, microvesicles, or direct cell-cell interaction, needs to be further specified. Yet, this stimulatory paracrine function may contribute to beneficial effects in applications such as ischemia, liver, and heart diseases. Furthermore, several studies have demonstrated that human MSCs reduce allorecognition, interfere with dendritic cell and T-cell function, and generate a local immunosuppressive microenvironment by secreting cytokines. This immunomodulatory function paved the way for cellular therapy in autoimmune diseases such as systemic lupus erythematosus, multiple sclerosis, or Crohns disease. Preliminary results with MSCs are promising for the treatment of graft-versus-host disease (GVHD) after allogeneic hematopoietic stem cell transplantation.

Although we are only starting to understand the mechanism of repair, the ease of culture isolation of MSCs, their moderate side effects in ongoing trials, and their pleiotropic functions make them good candidates for cellular therapy. There is an urgent need for further randomized, double-blinded, placebo-controlled clinical trials to unequivocally demonstrate safety and efficacy of MSCs. These results will also feedback on basic research to optimize culture conditions and cell preparations for a given application. This special issue summarizes review papers and clinical trials to provide insight in clinical perspectives of MSCs.

Several methods for isolation of MSCs from human bone marrow (BM) have been described: they are commonly isolated from the mononuclear cell fraction upon density gradient centrifugation. Alternatively, MSCs can be isolated by direct plating of BM. K. Mareschi and coworkers have compared these isolation regimens in “*multipotent mesenchymal stromal stem cell expansion by plating whole bone marrow at a low cellular density: a more advantageous method for clinical use*”. The results demonstrate that plating of whole bone marrow provides a suitable alternative for isolation of MSCs with relatively little hematopoietic contamination and slightly longer telomeres at first passage. Furthermore, the authors have addressed the impact of seeding density. Overall, this study supports the notion that MSCs have a diverse repertoire of distinct subpopulations which need to be taken into account. 

Alternatively, MSCs can also be isolated from adipose tissue (AT). These cells play a role in autologous lipotransfer for soft tissue reconstruction, and they can also be culture isolated. The anatomical location and the harvesting method may influence AT-MSCs and this has been addressed by M. Aguena and coworkers in “*Optimization of parameters for a more efficient use of adipose-derived stem cells in regenerative medicine therapies*”: comparison of samples from the lower abdomen *versus* flank revealed a significantly higher number of nucleated cells and expression of MSC markers in samples from the abdomen. Comparison of either pump-assisted liposuction or manual lipoaspiration did not affect cell numbers. These results exemplify the crucial role of starting material and cell isolation methods.

BM-derived MSCs have been described now over decades with regard to their osteogenic capacity. In this issue E. Zomorodian and M. Eslaminejad give an actual overview about “*Mesenchymal stem cells as a potent cell source for bone regeneration*”. First, the authors give a short summary about MSC from different tissues. But regardless of which source, osteogenic differentiation of MSC *in vitro* always has to be induced by inductive factors. Although many exogenous osteoinductive reagents have been described, sometimes the specific molecular pathways by which the cellular differentiation processes are modulated still need to be clarified. In addition, a better understanding of the *in vivo* migration of MSC to defect sites might improve their therapeutic use for bone repair strategies in the future. Another future prospect might be the application of MSC as vehicles for bone gene therapy, but also in this field many issues have to be solved. 

M. Mazo and colleagues review in “*Mesenchymal stem cells and cardiovascular disease: a bench to bedside roadmap*” the promising actions of MSC on injured myocardium by paracrine activity as well as differentiation into cardiovascular cell lineages. This comprehensive review paper demonstrates advantages of cellular therapy. However, the authors also point out that there are still many open questions at the level of basic research and animal models as well as even more at the outcome of clinical studies.

N. Venkataramana and coworkers have demonstrated in “Bilateral transplantation of allogenic adult human bone marrow-derived mesenchymal stem cells into the subventricular zone of parkinson's disease: a pilot clinical study” the safety of the procedure in 12 patients one year after the intervention. The authors assume beneficial neuroprotective and neurorestorative effects of MSCs. However, mixed results were obtained in this study, and only some patients showed a clinical improvement. This might be due to the fact that the duration of the disease varied widely in the study group. In addition, profound differences in the observed cell properties were mentioned, although the MSCs were only isolated from three different donors. Again, this clarifies that more basic work has to be done to enable a better definition of MSC (sub-) populations for a stable and reliable transplantation procedure in clinical settings.

MSCs mediate immunomodulatory effects. It might be possible that not only one subset of naïve stem cells but almost all mesenchymal stromal cells exhibit this immunogenic capacity. For example it has been demonstrated that mesenchymal stromal cells in general inhibit T-cell function. The development of GVHD is caused by T-cell reactivity. Kuzmina and coworkers contribute to this issue with the paper *“Multipotent mesenchymal stromal cells for the prophylaxis of acute graft-versus-host disease: a phase ii study*”. In this clinical study 19 patients received the standard GVHD prophylaxis with immunosuppresive in combination with the infusion of the MSCs of the hematopoietic stem cell donor during leucocyte recovery by activation of the hematopoietic transplant. This group was compared to 19 patients who were treated with the GVHD standard prophylaxis alone. In the MSC group only one patient developed acute GVHD, while in the standard group 6 patients suffered from this life-threatening disease. No differences in the graft rejection rates or in the incidence of infections were observed in both groups. But the overall mortality was 22.2% in the standard prophylaxis group compared to 5.3% in the MSC-treated group.

Taken together, MSCs provide promising perspectives for clinical applications with enormous potential for development, but the definite areas of application need to be further specified and validated. At the same time, a better molecular understanding is required for quality control and standardization of cellular therapeutics. 

*Jan Kramer* *Jan Kramer* *Francesco Dazzi* *Francesco Dazzi* *Massimo Dominici* *Massimo Dominici* *Peter Schlenke* *Peter Schlenke* *Wolfgang Wagner* *Wolfgang Wagner*

## Figures and Tables

**Figure 1:**
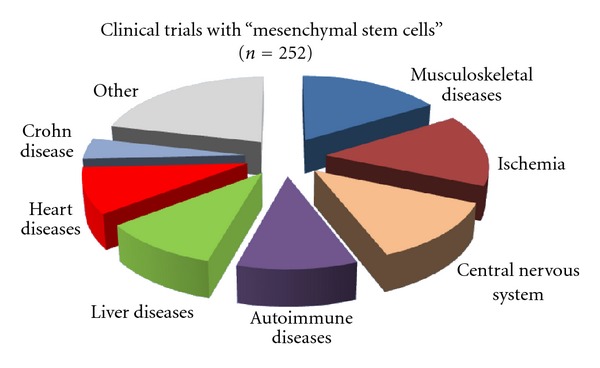
MSCs in clinical trials. 252 trials have been registered at http://www.clinicaltrials.gov/ (including 46 in North America, 61 in Europe, and 92 in East Asia, assessed on 11/20/2012). MSCs are tested for a broad range of diseases, and selected categories are presented.

